# Review of psychological stress on oocyte and early embryonic development in female mice

**DOI:** 10.1186/s12958-020-00657-1

**Published:** 2020-10-13

**Authors:** Qiu-Yue Zhai, Jun-Jie Wang, Yu Tian, Xiaofang Liu, Zhenhua Song

**Affiliations:** 1 School of Basic Medicine, Qingdao University, Qingdao, 266071 China; 2 Qingdao Medical College, Qingdao University, Qingdao, 266071 China; 3 College of Life Sciences, Institute of Reproductive Sciences, Qingdao Agricultural University, Qingdao, 266109 China; 4 Yellow Sea Fisheries Research Institute, Chinese Academy of Fishery Sciences, Qingdao, 266071 China

**Keywords:** Psychological stress, Oocyte, Early embryonic development, Subfertility

## Abstract

Psychological stress can cause adverse health effects in animals and humans. Accumulating evidence suggests that psychological stress in female mice is associated with ovarian developmental abnormalities accompanied by follicle and oocyte defects. Oocyte and early embryonic development are impaired in mice facing psychological stress, likely resulting from hormone signalling disorders, reactive oxygen species (ROS) accumulation and alterations in epigenetic modifications, which are primarily mediated by the hypothalamic-pituitary-adrenal (HPA) and hypothalamic-pituitary-ovarian (HPO) axes. The present evidence suggests that psychological stress is increasingly becoming the most common causative factor for female subfertility. Here, we review recent progress on the impact of psychological stress on female reproduction, particularly for oocyte and early embryonic development in female mice. This review highlights the connection between psychological stress and reproductive health and provides novel insight on human subfertility.

## Introduction

Psychological stress in humans usually refers to uncomfortable ‘emotional experiences’ accompanied by predictable biochemical, physiological and behavioural changes or responses [1]. Generally, when stressful events from the environment exceed adaptive capacity, people are thought to suffer from psychological stress. Statistics have shown that approximately 20–25% of patients with severe psychological stress go on to develop depression [1–3]. In addition, an underlying connection between psychological stress and some diseases, including repression, cardiovascular disease (CVD), human immunodeficiency virus (HIV)/Acquired Immune Deficiency Syndrome (AIDS) and cancer has been reported [4]. Thus, psychological stress has clinically been deemed a potential factor that threatens human health and should be given more attention.

There are two classes of psychological stress, acute and chronic, which can also be divided into disconnected and persistent psychological stress [5, 6]. As reported, a series of physiological responses involved in the nervous, endocrine, and immune systems are triggered in humans or animals when subjected to psychological stress [7, 8]. The hypothalamic-pituitary-adrenal (HPA) axis, a neuroendocrine regulatory network involved in controlling the response to stress and regulating many physiological activities, shows dysfunction in most patients with depression [9, 10]. Corticotrophin-releasing hormone (CRH) produced by the hypothalamus is the principal component of the HPA axis, and together with arginine vasopressin, regulate the stress response [7, 8, 11, 12]. Moreover, both innate and adaptive immune responses have been shown to be impaired by psychological stress [8], and immune recovery rates are delayed in humans with high levels of perceived psychological stress compared to those with low levels [13]. Together these results suggest that psychological stress is a potential threat to homeostasis throughout the body.

Presently, growing evidence suggests that psychological stress is involved in a series of biological events that place strain and pressure on systems that may cause certain reproductive system diseases [14–16]. Investigations into psychological stress and the female reproductive system have focused primarily on fertility and teratogenicity. For example, women suffering from stressful lives often also have reproductive disorders [17]. In addition, evidence has demonstrated that psychological stress often affects the number of retrieved and fertilized oocytes, as well as pregnancy, live birth delivery, birth weight and multiple gestations [15, 18]. A prospective study proposed that psychosocial stress in women could alter cortisol excretion patterns of the oestrous cycle and elevate the circulating prolactin level [19], which possibly affects the hormonal profile of the reproductive process, ultimately impacting fertility. Furthermore, that women suffering from psychological stress are at an increased risk of giving birth to preterm infants with low birth weight, and impaired reproductive capacity [19, 20]. Recent laboratory studies have shown that subjecting female mice to psychological stress during follicular growth and maturation significantly diminishes the developmental competence of oocytes, leading to follicular maldevelopment, ovulatory and luteal dysfunction [21]. Tan and colleagues indicated that restraint stress triggered chromosome aneuploidy by impairing spindle assembly checkpoint proteins in mouse oocytes [22]. Mice and rats exposed to restraint stress (a widely used experimental procedure for psychological stress) during gestation caused luteolysis, decreased serum progesterone (P4) concentrations and average litter size, retarded foetal development, and even a reduced pregnancy rate [23–25]. Moreover, the foetal growth and gestation length of ewes with short-term maternal psychological stress are affected, and research on sows has also demonstrated that regrouping of animals raised in groups after weaning can lead to stress and ultimately increase the risk of rebreeding [26, 27].

As highlighted above, a large amount of studies has indicated a certain causal relationship between psychological stress and female reproduction; however, the mechanisms underlying the effects of psychological stress on female reproduction are far from fully understood. Here, we focus on research advances of psychological stress on oocyte and early embryonic development in female mice, as well as the mechanism by which this psychological stress impacts female reproductive development and fertility.

### Overview of oogenesis and early embryonic development

**Fig. 1:**
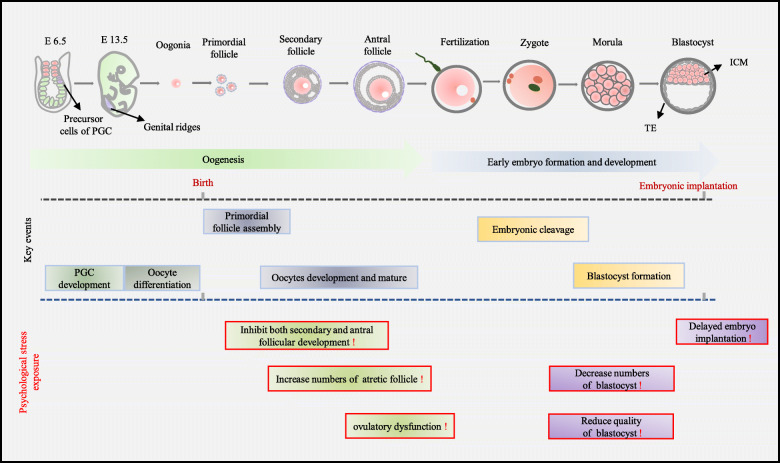
Schematic diagram of the main events and psychological impact on mouse oogenesis and early embryonic development. The process of oogenesis and early embryonic development was divided into oogenesis and early embryo formation and development. Psychological stress altered the balance of key events as depicted by the red frames in the bottom of the diagram. Primordial germ cells (PGC), precursor cells (E6.5) and genital ridges (E13.5) are shown in mauve

Generally, the embryo becomes implanted into the proximal endoderm and initiates early development. Around embryonic day 6.5 (E6.5), some ectodermal cells transform into the precursor cells of primordial germ cells (PGCs) [28]. With the inhibition of somatic cell expression module, the precursor cells of PGCs complete the specification process, subsequently migrate into the hindgut endoderm, and ultimately colonize the genital ridges (~E10.5). Following embryonic development, PGCs gradually enter meiosis and differentiate into oogonia at approximately E13.5, some of which end up with primordial follicles. Most of these primordial follicles are kept dormant, with only a fraction recruited to activate the growth process driven by bidirectional communication between the oocytes and the surrounding somatic cells [29], after which the primordial follicles further develop into secondary follicles and finally antral follicles [30]. The production of antral follicles largely depends on the secretion of multiple sex hormones, including gonadotropin-releasing hormone (GnRH), follicle-stimulating hormone (FSH), luteinizing hormone (LH) and oestrogens (E2) [31]. Most of the follicles undergo atretic degeneration, and only a subset of antral follicles (known as the dominant follicles) are ready to ovulate, which is triggered by LH. In addition, the granulosa and theca cells form the corpus luteum secrete P4 to maintain pregnancy [32, 33] (Fig. 1, oogenesis).

In the mouse, fertilization is achieved when a haploid sperm merges with an oocyte to form a diploid zygote, which is the starting point of new life [34]. Following fertilization in the oviduct, the zygote grows to a morula, which consists of approximately 8–16 cells after several cycles of mitotic divisions [35, 36]. The morula enters the uterine lumen and transforms into a blastocyst, which could be divided into two distinct layers: the inner cell mass (ICM) layer, i.e., a cluster of pluripotent cells attached to one inner side of the trophectoderm; and the outer epithelial cell layer, known as the trophectoderm (TE) (the precursor of trophoblast cells) [31] (Fig. 1, early embryo formation and development). Subsequently, embryos mature and escape from the zona pellucida, acquire implantation competence, and start post-implantation development.

### Effects of psychological stress on oocyte development and maturation

Currently, the chronic unpredictable mild stress (CUMS) model is widely accepted for psychological stress studies, especially in oocytes and early embryonic development. The CUMS paradigm consists of a series of mild and unpredictable stressors, including restraint, isolation or crowded housing, food or water deprivation, disruption of the dark-light cycle, and dampened bedding. Although different patterns of stress responses were triggered by various responses in animals, no invasive physical procedure or tissue trauma was allowed in psychological stress models [37–41]. Importantly, many researchers have demonstrated that CUMS leads to long-lasting changes in behavioural, neurochemical, neuroimmune and neuroendocrinological activities, which resemble dysfunctions observed in depressed patients [42].

Oocytes are largely influenced by psychological stress. The number of atretic antral follicles increased in mice under psychological stress, which was accompanied by impaired follicular development due to a lack of corpus luteum, pyknotic granulosa cells, and an irregular oestrous cycle [43]. Tan and colleagues investigated the effects of psychological stress on female mice using two methods, repeated restraint stress for 23 days or CUMS twice a day for 4 days, and found that the degree of damage to the oocytes following psychological stress was related to the duration and severity of the stress. Moreover, the accumulating effects of stress impair the developmental potential of oocytes, and the oocytes in preantral follicles are less sensitive than those in antral follicles [44]. Maternal separation (MS) as a model of early life stress causes a certain degree of psychological stress for offspring. The developmental parameters of in vitro cultured pre-antral follicles (PF), such as survival, growth, formation of antrum cavity, ovulation, as well as oocyte maturation were affected. This study also reported that psychological stress decreased follicular development by altering the oxidative status, which might lead reduced adult reproductive potential [45].

Using different stress animal models, psychological stress was found to inhibit both secondary and antral follicular development, as well as increase follicular atresia, which may be due to the suppressed expression of growth and differentiation factor 9 (GDF9) in mice [38]. Similarly, additional studies reported that the expression of brain-derived neurotropic factor (BDNF, a stress-responsive intercellular messenger protein) in antral follicles is reduced when mice are exposed to psychosocial stress [46].

Germinal vesicle breakdown (GVBD) is the key event conferring oocyte quality [47]. Following a 4-week psychological stress treatment, oocytes with abnormal metaphase II spindles (MII spindles) and non-surrounded nucleolus type (NSN-type) were increased, and the progression into M phase were delayed, suggesting that psychological stress compromised the meiotic competence of oocytes [41]. In addition, the percentage of surrounded nucleolus (SN) oocytes were significantly decreased during in vitro culture when female mice were subjected to psychological stress for 2 or 23 days [48]. Another investigation demonstrated that oocyte aneuploidy at MII stage was higher in maternal mice exposed to psychological stress, which was caused by impaired metaphase I (MI) spindle assembly [22].

Rat studies revealed that psychological stress decreased the number of secondary and antral follicles [49]. Rats in the CUMS groups showed irregular oestrous cycles caused by marked changes in the antral follicles and atretic follicles, which was partly attributed to BDNF-mediated PI3K/Akt pathway regulation [50]. Interestingly, more cystic follicles were found in the ovaries after similar psychological stress lasted for 12 weeks, suggesting that psychological stress may lead to polycystic ovary (PCO) [51]. Wang et al. reported that CUMS may cause premature ovarian failure (POF) in female rats. Thus, the CUMS model may be suitable for further assessment of the pathogenesis of psychological stress-induced POF [52] (Fig. 1, key events, and psychological stress exposure).

### Effects of psychological stress on early embryonic development

Given the sensitive nature, an early embryo is more vulnerable to prenatal stress than at later stages [53]. According to previous reports, restraint stress in mice impairs the blastocyst activation and hatching, as well as uterine receptivity in a time-dependent manner, and implantation impairment caused by stress is closely connected to both the embryo and the uterus [54]. Even if more embryos could be found in the oviducts, only a few embryos could reach blastocyst stage in pregnant mice exposed for 5 h per day to restraint for 3 days to model psychological stress, and with the number of implantation sites were much less when the mice were restrained for 6 days [23]. Using immobilization restraint, another supporting study revealed that mice exposed to psychological stress had a 40% reduction in the number of implantation sites and fewer delivered litters than controls [55]. Likewise, psychological stress that occurs during pregnancy influenced the developmental capacities of embryos, delayed blastocyst hatching, and even implantation failure [54], and one of those studies further revealed that psychological stress exposure decreased the number of ICM and TE cells in mouse blastocysts unevenly, and resulted in a higher ICM/TE ratio [56, 57]. Less retrieved oocytes, a lower fertilization rate, and less 2-cell embryos and blastocysts were detected in female mice when exposed for 4 weeks to nine stressors. This study also demonstrated that heat shock proteins 70 (HSP70), a cellular stress protein, plays a protective role in mice subjected to CUMS [58]. Additionally, maternal restraint stress at the oocyte pre-maturation stage resulted in higher percentages of aneuploid 2- to 4-cells, which subsequently led to lower percentages of parthenotes developing to either 4-cell or blastocyst stages [22]. In vivo embryo development of oocytes derived from mice restrained for 23 days showed that both the number of blastocysts obtained per mouse and the cell counts per blastocyst were significantly lower, and the average number of young per recipient decreased after embryo transfer compared to unstressed mice [48]. An in vitro study by Liu and collaborators provided evidence that the blastocyst formation rates in psychologically stressed mice are significantly decreased [46].

Applying a novel psychological stress model (mice were placed in oblong micro-cages in which they could not turn around) also resulted in reduced rates of blastocyst formation and cell counts per blastocyst both in vitro and in vivo. Notably, the number of young and their birth weights from stressed donors decreased significantly compared to those of control donors after embryo transfer [21], which was consistent with the results observed in mice exposed to natural predators [20]. Furthermore, systematic studies have shown that intrauterine growth retardation, disrupted organogenesis, faulty sex differentiation and external malformations were found in pregnant mice subjected to severe restraint procedures during early pregnancy [44]. Additional evidence suggests that the activation of the Fas system is required for detrimental effects to embryo development, as well as apoptosis in oviducts and embryos triggered by preimplantation restraint stress (PIRS) in mice. Increased tumour necrosis factor alpha (TNF-α) levels in oviducts and embryos implies that proinflammatory cytokines are also involved in apoptosis [59] (Fig. 1, key event, and psychological stress exposure).

### The mechanisms of psychological stress on oocyte and early embryonic development in female mice

#### Hormone signalling disorders caused by psychological stress

Existing studies have shown that the HPA and HPO axes are involved of the effects of psychological stress on oocytes and early embryonic development. Generally, the activation of HPA and HPO axes activation triggers gamma-aminobutyric acidergic (GABAergic) signalling by stimulating CRH neurons and GnRH neurons, respectively, in the paraventricular nucleus (PVN) of the hypothalamus [60], which increases the synthesis and release of neurosteroids, some of which are positive allosteric modulators of GABAA receptors (GABAARs), such as allopregnanolone and tetrahydrodeoxycorticosterone (THDOC). Overall, alterations of GABAergic signalling and neurosteroid levels are largely responsible for neuroendocrine dysfunction and enable vulnerability to psychological stress [61, 62].

**Fig. 2:**
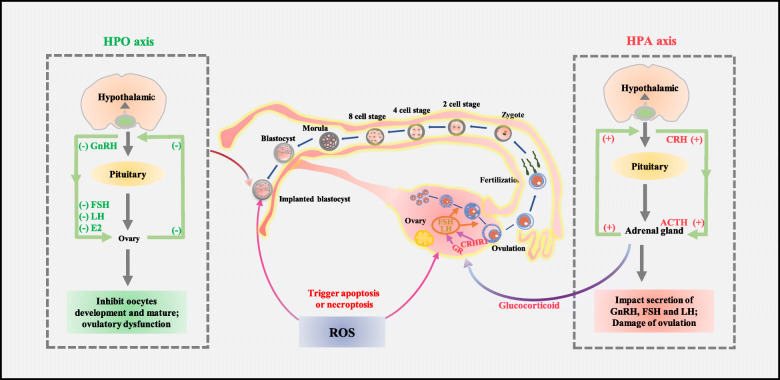
Psychological stress regulatory mechanism. Hormonal regulation resulting from psychological stress can be categorized into the HPA and HPO axis pathways. The HPA axis is involved in the neuroendocrine response to psychological stress (right panel). CRH locates the core regulator along the axis, which is released from the hypothalamus, stimulates ACTH release from the pituitary and glucocorticoid secretion from the adrenal gland. In the HPO axis (left panel), GnRH binds to anterior pituitary gonadotrophs to stimulate LH and FSH secretion. FSH acts on granulosa cells, while the LH receptor is primarily expressed by internal theca cells in response to LH stimulation. Decreased serum levels of E2 inhibit GnRH secretion via feedback to the hypothalamus and pituitary gland. The middle panel shows the main biological events in embryonic development and oogenesis. ROS affect oocyte development and maturity mainly through targeted apoptosis and necroptosis

In terms of HPA-mediated signalling pathways, CRH neurons activated in the hypothalamus can stimulate the pituitary to synthesize and secrete adrenocorticotropic hormone (ACTH), which subsequently induces glucocorticoid production (corticosterone in mice) by the adrenal cortex [63]. Increased glucocorticoid levels are also responsible for regulating the HPA axis via a negative feedback loop and terminating the stress response (Fig. 2, the HPA axis). Proper gonadal function is disrupted because of glucocorticoid imbalance, which consequently has serious implications for fertility [64].

As reported, glucocorticoids affect ovarian function in multiple ways. The primary mechanism is through inhibiting the synthesis and release of GnRH in the hypothalamus, which further results in reduced LH and FSH in the pituitary. Glucocorticoids are mainly present in hypothalamic neurons and ovaries, which can affect the production of testosterone by regulating the glucocorticoid receptor (GR) or directly inducing apoptosis, thereby further affecting female reproductive function [65]. In addition, since the ovary does not locally produce glucocorticoids, other actions of glucocorticoids in the ovary are mediated by 11β-hydroxysteroid dehydrogenase (11β-HSD), and the activation of 11β-HSD can catalyse both oxidative and reductive reactions during follicular maturation [66].

Numerous studies have examined the effects of glucocorticoids on reproductive function. Mouse serum cortisol and corticosterone (two biomarkers for stress and depression) levels are closely correlated in terms of their dynamic levels throughout the oestrous cycle [39]. In stressed mice, cortisol levels were elevated, and 63% of mice showed irregular cycling, whereas 37% were found to be acyclic. These results support the idea that anovulation caused by psychological stress may be due to increased cortisol levels [37]. Further investigations found that cortisol exposure leads to decreased GR expression and increased Fas expression in mural granule cells, which indicated that cortisol-triggered mural granulosa cell apoptosis was related to Fas activation. Prior studies also demonstrated that increased cortisol reduces growth factor levels and the E2/P4 ratio. Taken together, glucocorticoids have been found to diminish the developmental potential of oocytes by triggering ovarian cell apoptosis via the Fas system [67]. Hence, the HPA axis along with a corresponding inhibition of the HPO may underlie the effect of psychological stress on female reproduction.

How does the HPO axis affect reproductive development when psychological stress occurs? Like the HPA signalling pathway, when the body is under psychological stress, CRH can inhibit the secretion of GnRH in the hypothalamus, thereby reducing the synthesis and release of FSH and LH in the pituitary (Fig. 2, the HPO axis). Lower FSH and LH levels reflect the fact that E2 and P4 secretion in the ovary is impeded [68]. Indeed, the two fundamental functions of the ovary are producing oocytes and secreting steroid hormones that are necessary for fertilization and embryo implantation. In mammals, many factors are involved in regulating ovarian development, but FSH and LH have a central role in endocrine mechanisms [69]. Specifically, FSH receptors are located at the granulosa cells, while LH receptors are primarily expressed on internal theca cells, both of which are crucial for E2 synthesis through the cooperation of the two cell types. Follicular development is a continuing process that can be divided into initial follicle development, FSH-dependent progression, and LH-responsive maturation. Although FSH and LH play major roles in different time windows, FSH in synergy with LH plays an obligatory role in normal follicle differentiation, growth, selection, survival, and ovulation [69–71].

In addition, CRH and its receptors have been identified in several tissues of the female reproductive system, including the ovaries, endometrium, and placenta [72–74]. Ovarian CRH, primarily found in the theca, stroma, and cytoplasm of ovum and granulosa cells, can regulate follicular maturation, ovulation, luteolysis and ovarian steroidogenesis. Disruption of CRH expression leads to premature ovarian, anovulation, corpus luteum and ovarian dysfunction [72–75]. At the uterine level, CRH has effects on decidualization and blastocyst implantation, and decreased CRH secretion causes infertility and recurrent spontaneous abortion [72]. Moreover, abnormal placental CRH levels have been demonstrated to impair labour, fetoplacental circulation and foetal adrenal steroidogenesis as well as triggering maternal hypercortisolism, and premature and delayed labour [72].

Some evidence has suggested that CRH modifies the HPO response and subsequently leads to reproductive failure in psychologically stressed mice. For example, after female mice were subjected to restraint stress for 24 h, the CRH levels in the serum, ovary and oocytes were increased, and the number of apoptotic cumulus cells (CCs) and MGCs after in vitro maturation (IVM) were significantly higher, indicating that oocyte incompetence caused by psychological stress may be due to the latent apoptotic programme initiated by increased CRH levels. Moreover, the more CRH that interacts with the CRH receptor 1 (CRHR1) on thecal cells and MGCs, the greater the possibility of an imbalance between E2 and P4 by decreasing the availability of insulin-like growth factor 1 (IGF1) and BDNF, and the greater the possibility that oocyte competence will be eventually reduced. CRH also reduced blastocyst formation rates, and supplementation with the CRHR1 antagonist antalarmin further confirmed the results in CCs [76].

#### Accumulation of ROS under psychological stress

Apart from hormonal imbalance caused by psychological stress, the compromised antioxidant status also adversely affects ovarian function due ROS accumulation [77]. Studies have shown that the physiological level of ROS may be beneficial in folliculogenesis, oocyte maturation, ovarian steroidogenesis and luteolysis [78–80], but ROS accumulation beyond the physiological level could result in oxidative stress (OS) that is harmful to female reproduction. Generally, the ROS produced by psychological stress impacting reproductive outcomes is likely mediated by various pathways. To be specific, ROS can diffuse and pass through cell membranes and alter most types of cellular molecules such as lipids, protein, and nucleic acids, induce mitochondrial alterations, deplete ATP, increase the rate of DNA fragmentation in the nucleus, and trigger apoptosis and necrosis in most granulosa cells, oocytes, and even early embryos. Additionally, increased ROS in the microenvironment of the ovary, fallopian tube, and uterus lead to changes in antioxidant enzyme levels, which are important in protecting oocytes and early embryo development. ROS also results in reproductive hormone regulation imbalances, which results in oocyte damage and early development and maturity [81, 82].

Recent studies have indicated that the generation of OS because of ROS accumulation, which reduces oestradiol-17β biosynthesis in the ovaries, triggers apoptosis in most granulosa cells and disrupts the developing follicular oocytes [78]. OS can also trigger necroptosis, an unregulated cell death, in granulosa cells and follicular oocytes [83]. Excessive TNF-α attenuated ovarian development by reducing the number of mature follicles and disrupting oocyte meiotic maturation in mice [84]. Specifically, excessive TNF-α binds to its receptor (TNFR) on the granulosa cell and oocyte membranes, which triggers necroptosis through the receptor interacting protein kinase-1 (RIPK1)-mediated pathway [85], and ultimately may cause follicular atresia and infertility [86]. Furthermore, ROS can negatively affect early embryo implantation and may influence the development of reproductive disorders by modifying key transcription factors and hence gene expression [87]. Psychological stress during preimplantation impaired embryo development via the Fas system and caspase-3 signalling, which was accompanied by a significant increase in OS markers in the serum, oviducts and embryos, indicating that psychological stress triggered apoptosis through OS activity [59]. Additionally, OS also induces telomere shortening, chromosomal segregation disorders, oocyte fragmentation and fertilization failures [88, 89]. Research in male mice also showed that an overabundance of ROS in sperm impacts DNA integrity, which altered the dynamics of epigenetic reprogramming and resulted in the progressive loss of the ability to repair DNA damage [90].

#### The impact of psychological stress on epigenetic modification

During mammalian development, epigenetic modification, including DNA methylation, histone modification and small non-coding RNA, is essential for early germ cell development and the generation of functional gametes [91, 92]. In physiological amounts, dynamic changes in DNA methylation and demethylation are crucial in the epigenetic regulation of mammalian gametic and embryonic development. When an organism suffers psychological stress, hormone regulation imbalances and increased ROS impact not only DNA integrity but also epigenetic reprogramming, which may eventually affect the development and quality of oocytes and embryos [93].

A substantial amount of evidence has revealed a causal connection between psychosocial stress events and epigenetic modifications [94, 95]. In experimental models, some offspring behaviours have been shown to be acquired from the mother under situations where the mother was exposed to psychosocial stress [96]. These results present the possibility that the impact of maternal psychological stress could be transgenerational through epigenetic modification on offspring germ cells. More recently, evidence from rodent studies suggest that risk factors from psychological stress can be transmitted to subsequent generations through epigenetic mechanisms, by altering DNA methylation and potentially contributing to a cycle of disease and disease risk [97]. At present, studies about the negative influence of maternal psychological stress during the pregnancy/perinatal period on the offspring’s brain and inter-generational inheritance has attracted much attention [93, 98]. These studies reported increased transplacental transfer of glucocorticoids from the mother to the foetus triggered by prenatal psychological stress, which increased GR gene transcription and affected the availability of methyl donors that alter DNA methylation patterns. These changes can impact brain development and the mental health of the foetus [99]. Interestingly, psychological stress in females inhibited the NSN-SN transition, and the presence of H3K14 and H4K12 acetylation, as well as H3K4me2 and H3K9me3 methylation in SN oocytes, was significantly lower in stressed versus untreated groups. These data provide evidence of psychological stress impairing the developmental potential of oocytes through epigenetic mechanisms [48]. Based on the existing evidence, we speculate that psychological stress may at least partially induce abnormal epigenetic modifications in oocytes, leading to the production of low-quality oocytes and ultimately influencing female reproduction.

## Conclusions

In this review, the effects and potential mechanisms of psychological stress on oocyte and early embryonic development in female mice are highlighted. Impacted oocyte development (including maturation and ovulation) and an increased number of atretic follicles were found in mice exposed to psychological stress, and embryonic development (including preimplantation/peri-implantation development) was affected, leading to lower implantation rates, teratogenic outcomes and even miscarriages. In terms of mechanism, psychological stress could affect the HPA and HPO axis, which are major pathways that alter the level of hormones and directly generate OS. Further, these axes indirectly induce apoptosis and necroptosis, ultimately reducing oocyte quality and the reproductive outcome of female mice. Moreover, the impact of epigenetic modification should never be neglected in maternal psychological stress models. In conclusion, animal studies have elucidated the adverse effects of psychological stresses on the neuroendocrine system and have provided novel insights into the mechanisms of ovarian dysfunction and infertility caused by psychological stress in humans.

## Data Availability

Not applicable.

## Abbreviations

ROS: Reactive oxygen species
HPA: Hypothalamic-pituitary-adrenal
HPO: Hypothalamic-pituitary-ovarian
CVD: Cardiovascular disease
HIV: Human immunodeficiency virus
CRH: Corticotrophin-releasing hormone
P4: Progesterone
ICM: Inner cell mass
TE: Trophectoderm
PGCs: Primordial germ cells
GnRH: Gonadotropin-releasing hormone
FSH: Follicle-stimulating hormone
LH: Luteinizing hormone
E2: Oestrogens
CUMS: Chronic unpredictable mild stress
HB-EGF: Heparin-binding epidermal growth factor-like growth factor
PIRS: Preimplantation restraint stress
TNF-α: Tumour necrosis factor
HSP70: Heat shock proteins 70
BDNF: Brain-derived neurotropic factor
GDF9: Growth and differentiation factor 9
GVBD: Germinal vesicle breakdown
MII-spindles: Metaphase II-spindles
NSN-type: No-surrounded nucleolus type
CCNB1: Cyclin B1
SN: Surrounded nucleolus
MI: Metaphase I
PCO: Polycystic ovary
POF: Premature ovarian failure
GABAergic: Gamma-amino butyric acidergic
PVN: Paraventricular nucleus
GABAARs: GABAA receptors
THDOC: Tetrahydrodeoxycorticosterone
ACTH: Adrenocorticotropic hormone
GR: Glucocorticoid receptor
11β-HSD: 11β-hydroxysteroid dehydrogenase
CCs: Cumulus cells
IVM: In vitro maturation
CRHR1: CRH receptor 1
IGF1: Insulin-like growth factor 1
OS: Oxidative stress
TNFR: TNF-α binds to its receptor
RIPK1: Receptor interacting protein kinase-1
